# Author Correction: A framework for in situ molecular characterization of coral holobionts using nanopore sequencing

**DOI:** 10.1038/s41598-021-81544-6

**Published:** 2021-01-18

**Authors:** Quentin Carradec, Julie Poulain, Emilie Boissin, Benjamin C. C. Hume, Christian R. Voolstra, Maren Ziegler, Stefan Engelen, Corinne Cruaud, Serge Planes, Patrick Wincker

**Affiliations:** 1 Génomique Métabolique, Genoscope, Institut François Jacob, CEA, CNRS, Univ Evry, Université Paris-Saclay, 91057 Evry, France; 2 Research Federation for the Study of Global Ocean Systems Ecology and Evolution, R2022/Tara Oceans GO-SEE, 3 rue Michel-Ange, 75016 Paris, France; 3 PSL Research University: EPHE-UPVD-CNRS, USR 3278 CRIOBE, Université de Perpignan, Perpignan Cedex, France; 4 Laboratoire D’Excellence “CORAIL”, 52 Avenue Paul Alduy, 66860 Perpignan Cedex, France; 5 Red Sea Research Center, Division of Biological and Environmental Science and Engineering (BESE), King Abdullah University of Science and Technology (KAUST, Thuwal, Saudi Arabia; 6 Department of Biology, University of Konstanz, Universitätsstraße 10, 78457 Konstanz, Germany; 7 Department of Animal Ecology and Systematics, Justus LiebigUniversity, Giessen, Germany; 8 Genoscope, Institut de biologie François-Jacob, Commissariat à l’Energie Atomique (CEA), Université Paris-Saclay, Evry, France

Correction to: *Scientific Reports* 10.1038/s41598-020-72589-0, published online 27 May 2020

The Acknowledgements section in this Article is incomplete.

“This project has been funded through the *Tara* Pacific consortium, France Genomique Grant Number ANR-10-INBS-09, and the Genoscope/CEA. We are keen to thank the commitment of the people and the following institutions and sponsors who made this singular expedition possible: CNRS, CSM, PSL, KAUST, Genoscope/CEA, ANR-CORALGENE, agnès b., the Veolia Environment Foundation, Region Bretagne, Serge Ferrari, Billerudkorsnas, AmerisourceBergen Company, Lorient Agglomération, Oceans by Disney, the Prince Albert II de Monaco Foundation, L′Oreal, Biotherm, France Collectivites, Kankyo Station, Fonds Francais pour l′Environnement Mondial (FFEM), Etienne Bourgois, UNESCO-IOC, the Tara Foundation teams and crew. Tara Pacific would not exist without the continuous support of the participating institutes. This publication is number 5 of the Tara Pacific Consortium.”

should read:

“This project has been funded through the *Tara* Pacific consortium, France Genomique Grant Number ANR-10-INBS-09, and the Genoscope/CEA. We are keen to thank the commitment of the people and the following institutions and sponsors who made this singular expedition possible: CNRS, CSM, PSL, KAUST, Genoscope/CEA, ANR-CORALGENE, agnès b., the Veolia Environment Foundation, Region Bretagne, Serge Ferrari, Billerudkorsnas, AmerisourceBergen Company, Lorient Agglomération, Oceans by Disney, the Prince Albert II de Monaco Foundation, L′Oreal, Biotherm, France Collectivites, Kankyo Station, Fonds Francais pour l′Environnement Mondial (FFEM), Etienne Bourgois, UNESCO-IOC, the Tara Foundation teams and crew. Tara Pacific would not exist without the continuous support of the participating institutes. We also thank Professor Russel Clark Perembo, Alfred Yohang Ko’ou, Augustine Mungkaje and Professor Simon Saulei, researchers of the Marine Scientific Research Committee and the National Research Institute of Papua New Guinea for the authorization of coral sampling and their valuable assistance during Tara expedition in Papua New Guinea. This publication is number 5 of the Tara Pacific Consortium.”

Additionally, the information about research permissions is not included in the Article. The sampling and research described in this paper was approved by the Marine Scientific Research committee and the department of foreign affairs and trade of the independent state of Papua New Guinea, under the diplomatic clearance number 0232.

