# Supplementary material for: A framework for in situ molecular characterization of coral holobionts using nanopore sequencing
Source: Sci Rep. 2020 Sep 28;10:15893. doi: 10.1038/s41598-020-72589-0 (PMC7522235; doi:10.1038/s41598-020-72589-0)
Supplement: Supplementary file 1 — Supplementary file1. [file 41598_2020_72589_MOESM1_ESM.pdf]

Supplementary Information

A framework for in situ molecular characterization of coral holobionts using nanopore sequencing.

Quentin Carradec[1,2,*,†], Julie Poulain[1,2,*], Emilie Boissin[3,4], Benjamin CC Hume[5], Christian R Voolstra[5,6], Maren Ziegler[7], Stefan Engelen[8], Corinne Cruaud[8], Serge Planes[2,3,4], Patrick Wincker[1,2,†]

Table of Contents:

**Supplementary Figure S1**: Pictures of sampled coral colonies	Page 2
**Supplementary Figure S2**: Maximum Likelihood phylogenetic tree of coral 18S rRNA sequences.	Page 4
**Supplementary Figure S3**: ITS2 variants detected in coral samples.	Page 5
**Supplementary Method S1**: Bioinformatic pipeline and requirements to the analysis of nanopore sequences.	Page 6

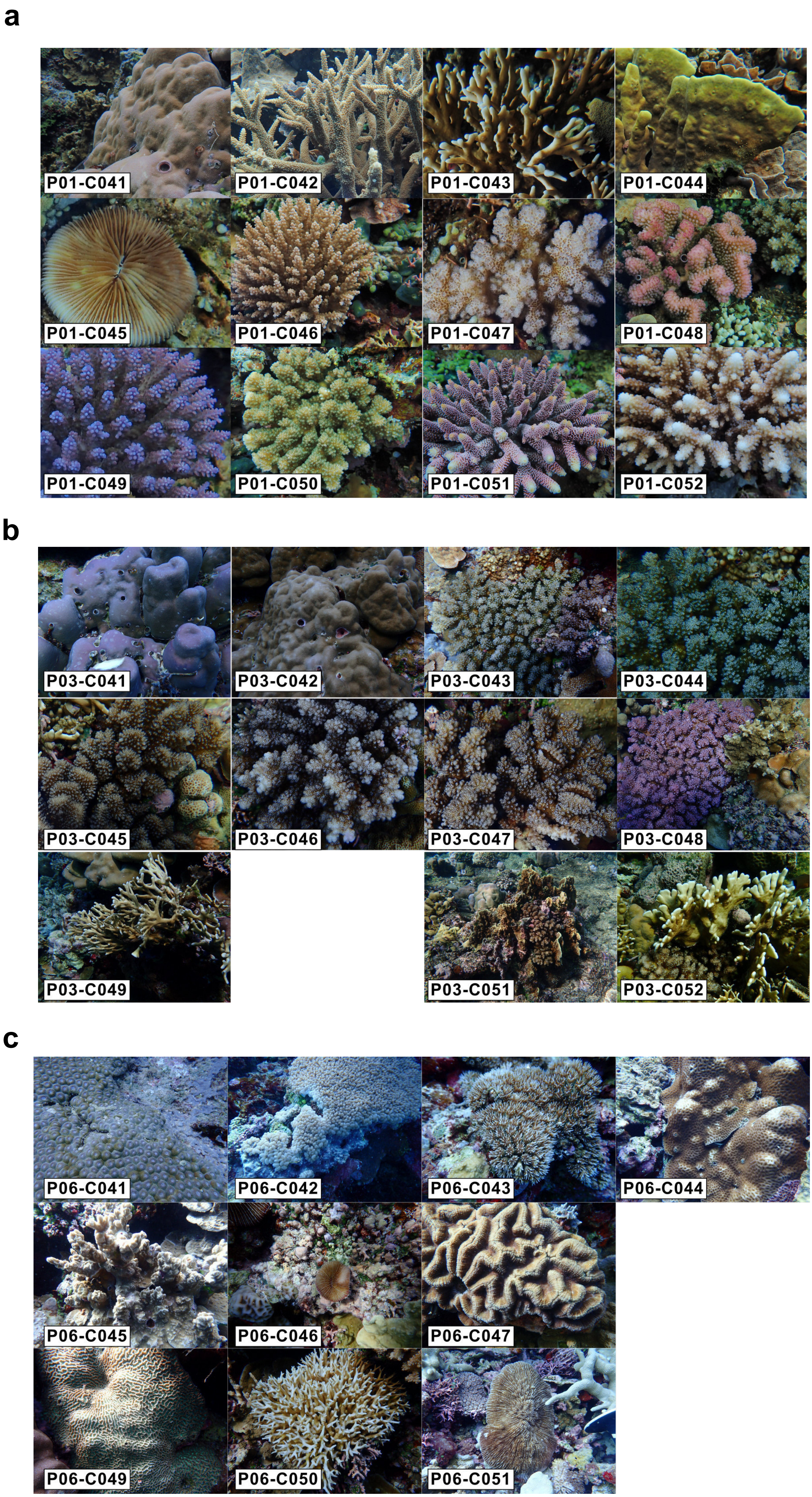
a
P01-C041
P01-C042
P01-C043
P01-C044
P01-C045
P01-C046
P01-C047
P01-C048
P01-C049
P01-C050
P01-C051
P01-C052
b
P03-C041
P03-C042
P03-C043
P03-C044
P03-C045
P03-C046
P03-C047
P03-C048
P03-C049
P03-C051
P03-C052
c
P06-C041
P06-C042
P06-C043
P06-C044
P06-C045
P06-C046
P06-C047
P06-C049
P06-C050
P06-C051

d

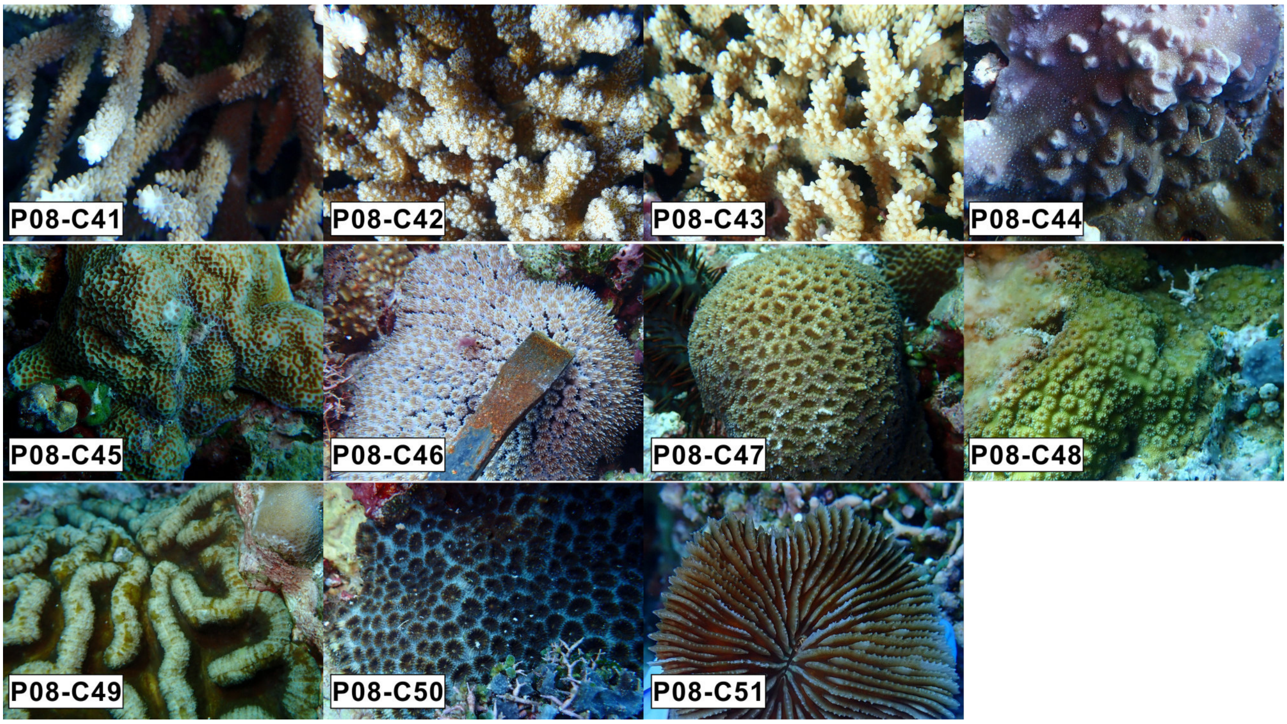

e

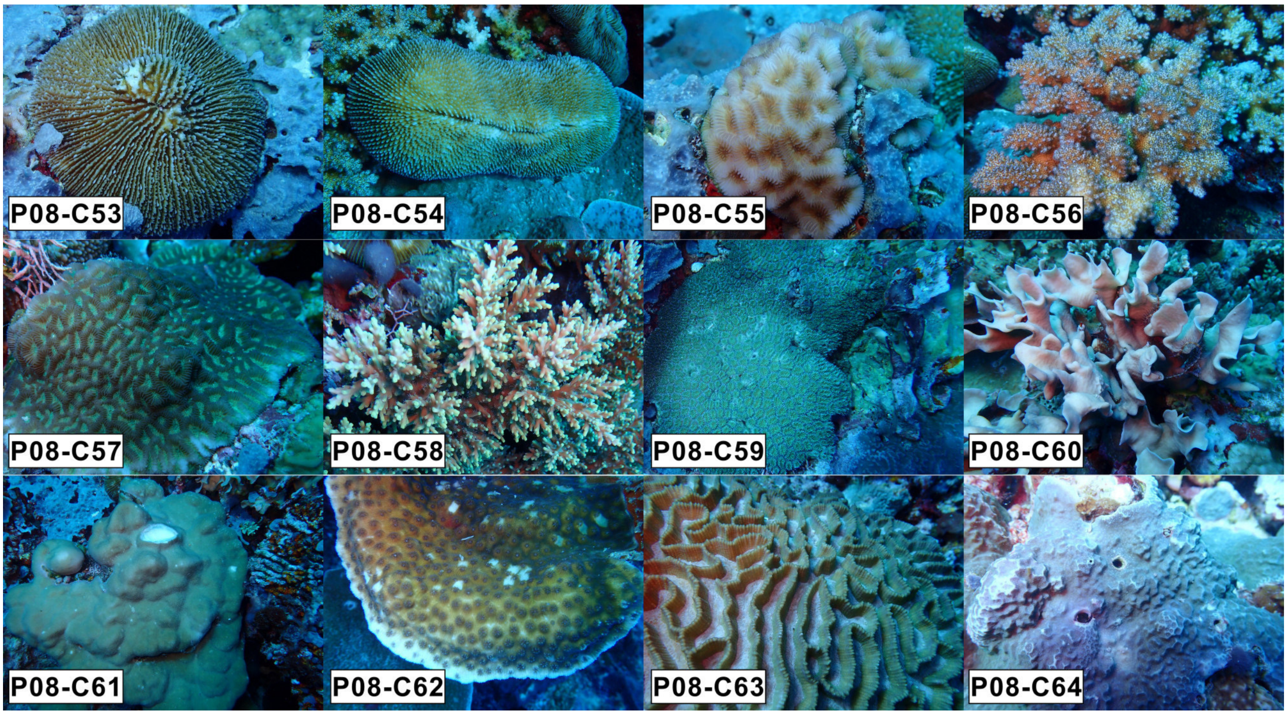

Supplementary Figure S1:Pictures of sampled coral colonies. a) P01-Run1. Porites lobata (C41), Acropora (C42), Millepora dichotoma (C43), Millepora platyphylla (C44), Fungia (C45), Pocillopora damicornis (C46), Acropora (C47), Pocillopora meandrina (C48), Pocillopora verrucosa (C49), Acropora (C50), Acropora (C51), Acropora (C52). **b)**. P03-Run2. Porites (C41), Porites lobata (C42), Pocillopora verrucosa (C43), Pocillopora verrucosa (C44), Pocillopora (C45), Pocillopora meandrina (C46), Pocillopora verrucosa (C47), Pocillopora (C48), Millepora dichotoma (C49), Millepora platyphylla (C51), Millepora dichotoma (C52). **c)** P06-Run3. Diploastrea (C41), Physogyra (C42), Galaxea (C43), Favites (C44), Porites rus (C45), Fungia (C46), Symphyllia (C47), Platygyra (C49), Seriatopora (C50), Fugiidae (C51). **d)** P08-Run4. Acropora (C41), Pocillopora verrucosa (C42), Acropora (C43), Porites rus (C44), Porites (C45), Galaxea (C46), Montastrea (C47), Cyphastrea (C48), Lobophyllia (C49), Favia (C50), Fungia (C51). **e)** P08-Run5. Fungia (C53), Fungia (C54), Pocillopora (C56), Goniastrea (C57), Acropora (C58), Galaxea (C59), Pavona cactus (C60), Porites Lobata (C61), Echinopora (C62), Oulophyllia (C63), Porites (C64).

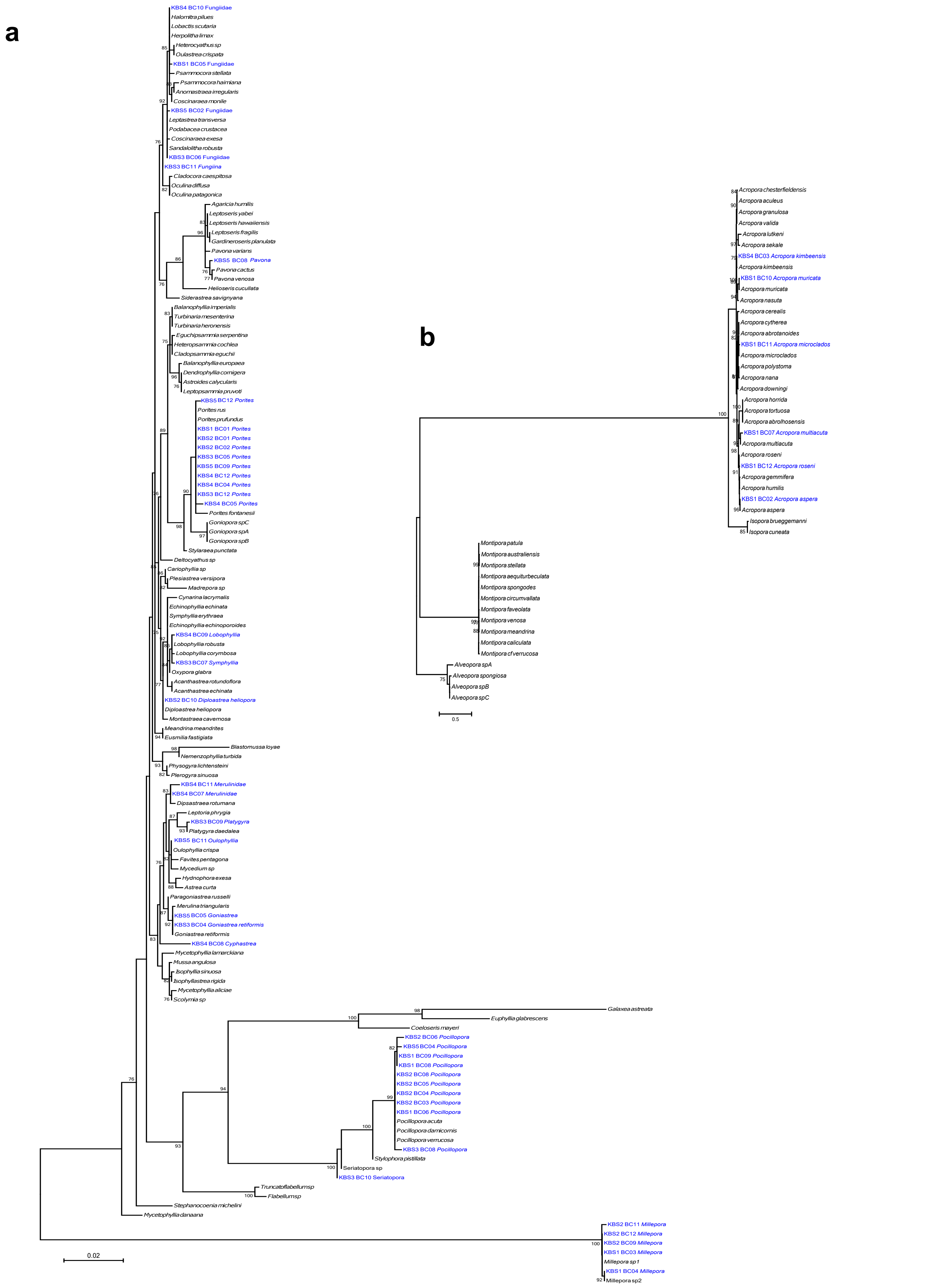

Supplementary Figure S2: Maximum Likelihood phylogenetic tree of coral 18S rRNA sequences. Sequences reconstructed in this study together with all 18S rRNA sequences from Arrigoni et al. 2018 are represented (12). (a) All corals except Acroporidae. (b) All Acroporidae 18S rRNA.

***Durusdinium* D1**

```
                  T(D10)
AACCAATGGCCCCCTGAACGCGCATTGCACTCTTGGGACTTCCTGAGAGTATGT
              G(D22)T(D17)
TTGCTTCAGTGCTTATTTTACCTCCTTGCAAGGTTCTGTCGCAACCTTGTGCCC
                                       G(InsD6)
TGGCCAGCCATGGGTTAACTTGCCCATGGCTTGCTGAGTAGTGATCTTTTAGAG
         C(D6,D2,D1a)
CAAGCTCTGGCACGCTGTTGTTTGAGGCAGCCTATATTGAGGCTATTTCAAATG

ACGTTGCTACAAGCTTGATGTGTCCTTCTGCGCCGTTGCGCATCCCATAGCATGA
                                          A(D1.6)
```

***Cladocopium* C40**

```
AACCAATGGCCTCCTGAACGTGCGTTGCACTCTTGGGATTTCCTGAGAGTATGTCTGCTT

CAGTGCTTAACTTGCCCCAACTTTGCAAGCAGGATGTGTTTCTGCCTTGCGTTCTTATGA
                  *(C3b)
GCTATTGCCCTCTGAGCCAATGGCTTGTTAATTGCTTGGTTCTGGCAAAATGCTTTGCGC
                          G(InsC40b)
GCTGTTATTCAAGTTTCTACCTTCGTGGTTTTACTTGAGTGACGCTGCTCATGCTTGCGA
                                                     (C3b)A
CCGCTGGGATGCAGGTGCATGCCTCTAGCATGAAGTCAGACAA
```

***Cladocopium* C66**

```
AACCAATGGCCTCCTGAACGTGCGTTGCACTCTTGGGATTTCCTGAGAGTATGTCTGCTT

CAGTGCTTAACTTGCCCCAACTTTGCAAGCAGGATGTTTTTCTGCCTTGCGTTCTTATGA
T(C74)
GCTATTGTCCTCTGCGCCAATGGCTTGTTAATTGCTTGGTGCTTGCAAAATGCTTTGCGC
   G(C57a)    T(C57C57a)
GCTGTTATTCAAGTTTCTACCTTCGTGGTTTTACTTGAGTGACGCTGCTCATGCTTGCAA
                                          T(C74)
CCCGCTGGGATGCAGGTGCATGCCTCTAGCATGAAGTCAGACAA
```

***Cladocopium* C15**

```
AACCAATGGCCTCCTGAACGTGCGTTGCACCCTTGGGATTTCCTGAGAGTATGTCTGCTT
               T(C19)         T(C2)
CAGTGCTTAACTTGCCCCAACTTTGCAAGCAGGATGTGTTTCTGCCTTGCGTTCTTATGA
               *(C19)
GCTATTGCCTTCTGCGCCAATGGCTTGTTAATTGCTTGGTTCTTGCAAAATGCTTTGCGC
    C(C60)         C(InsC15f)                          (C60)T
GCTGTTATTCAAGTTTCTACCTTCGCGGTTTTACTTGAGTGACGCTGCTCATGCTTGCAA

CCGCTGGGATGCAGGTGCATGCCTCTAGCATGAAGTCAGACAA
    T(InsC2)
```

Supplementary Figure S3: ITS2 variants detected in coral samples. ITS2 variants of D1, C40, C66 and C15, are indicated with a specific colour and their corresponding ITS2 sequence name. Ins= nucleotide insertion, *= nucleotide deletion.

Supplementary Method S1: Bioinformatic pipeline and requirements to the analysis of nanopore sequences.

Requirements:

- A Linux based operating system.
- Perl 5.8.0 or higher installed.
- Python 3.6.5 or higher
- Samtools 1.10.2 or higher
- Bcftools 1.10.2 or higher
- Porechop v 0.2.3
- Minimap2 v 2.0-r191

Analysis of nanopore reads and reconstruction of consensus sequences step by step:

1) Demultiplexing of nanopore reads.

```
PROJECTNAME="Project-name"

porechop --discard_middle -i Run_$PROJECTNAME.fastq -b Run_$PROJECTNAME.barcode -v 2 -t 4 > Run_$NAME.porechop.log
```

Steps 2 to 8 should be executed for each marker gene and for each barcode of the experiment. Barcode BC01 for ITS2 marker gene is given as an example.

2) First mapping with Minimap2 then conversion to BAM format.

```
FQ="Nanopore-reads_barcode01.fastq"

minimap2 --secondary=no -a -x map-ont $DB $FQ | samtools view -b -F 4 > ${FQ}_ITS2.bam
```

3) Selection of the reference sequences covered by at least 0.01% of all nanopore reads.

```
samtools view ${FQ}_ITS2.bam | awk '{print $3}' | sort | uniq -c | sort -nrk1,1| perl -e 'while(<>){chomp;split;$h{$_[1]}=$_[0];if($_[0]>=10){$t+=$_[0]}};foreach $k (keys %h){if($h{$k}>=0.01*$t){print "$k\n"}}' > ${FQ}_ITS2_BestRef.name
```

4) Sequence extraction of the selected references in the database.

```
samtools faidx $DB `cat ${FQ}_ITS2_BestRef.name` > ${FQ}_ITS2_BestRef.fa
```

5) 2nd round of mapping with Minimap2 then conversion to BAM format

```
minimap2 --secondary=no -a -x map-ont ${FQ}_ITS2_BestRef.fa $FQ | samtools view -b -F 4 > ${FQ}_ITS2_BestRef.bam
```

6) Calculation of the number of reads aligned on each reference

```
samtools view ${FQ}_ITS2_BestRef.bam | awk '{print $3}' | sort | uniq -c | sort -nrk1,1| perl -e 'while(<>){chomp;split;$h{$_[1]}=$_[0];$t+=$_[0]};foreach $k (keys %h){print "$k\t$h{$k}\t$t\n"}' > ${FQ}_ITS2_BestRef.stat
```

7) Reconstruction of the consensus sequence.

```
samtools sort ${FQ}_ITS2_BestRef.bam > ${FQ}_ITS2_BestRef.sort.bam
```

```
samtools mpileup -B -a -Q 0 -u -f $DB ${FQ}_ITS2_BestRef.sort.bam | bcftools call -c | vcfutils.pl vcf2fq > ${FQ}_ITS2_BestRef.consensus.fq
```
